# Enhanced Vernier Effect in Cascaded Fiber Loop Interferometers for Improving Temperature Sensitivity

**DOI:** 10.3390/s25010038

**Published:** 2024-12-25

**Authors:** Jianming Zhou, Yanyan Zhi, Junyi Zhang, Jianping Yao, Junkai Zhang, Jiejun Zhang

**Affiliations:** 1 Guangdong Provincial Key Laboratory of Optical Fiber Sensing and Communication, Institute of Photonics Technology, Jinan University, Guangzhou 510632, China; zhoujianming@stu2020.jnu.edu.cn (J.Z.);; 2 College of Physics & Optoelectronic Engineering, Jinan University, Guangzhou 510632, China

**Keywords:** microwave photonics, enhanced Vernier effect, optical fiber sensor, temperature sensor

## Abstract

This work presents a high-sensitivity temperature sensing system utilizing an enhanced Vernier effect implemented in cascaded fiber loop interferometers. High-sensitivity temperature sensors based on the Vernier effect have broad application prospects, but the sensitivity of traditional measurement schemes is difficult to improve further due to the limited variation in the difference between two free spectrum ranges (FSRs). Our sensing system incorporates two fiber loop interferometers and a single-mode fiber to form a Vernier spectral response, characterized by two complementary optical filter responses. As the temperature of the sensing fiber changes, one FSR decreases, and the other increases, respectively, enhancing the difference value between the two FSRs to form an enhanced Vernier effect. Experimental results demonstrate that the temperature sensitivity of a traditional Vernier effect measurement is only −298.29 kHz/°C, while our proposed enhanced Vernier effect sensing system achieves a sensitivity of 618.14 kHz/°C, which is 92 times higher than that of a two-arm optical carrier-based microwave interferometry (OCMI) sensing system and 2.07 times higher than that of a traditional Vernier effect sensing system. This approach with an enhanced Vernier effect scheme based on cascaded fiber loop interferometers can be used to design high-sensitivity sensing systems for biometrics, smart cities, and the Internet of Things.

## 1. Introduction

Highly sensitive temperature detections are widely used in industrial production fields such as lithium-ion battery monitoring [1], biomedical sensing [2], and oil and gas pipeline safety management [3]. Traditional electrical sensors for temperature measurements are often associated with high energy consumption and high costs. In contrast, advanced optical sensing technologies, such as Raman spectroscopy and optical fiber sensing, offer potential solutions to these challenges [4]. The Raman spectroscopy analysis technique utilizes the temperature-sensitive characteristics of Raman scattering and is widely used for high-precision, high-resolution detection in biochemical environments and food safety [5]. Optical fiber sensing technology, with its advantages of small size, low energy consumption, corrosion resistance, and immunity to electromagnetic interference, is widely used in sensing applications for temperature, pressure, and curvature [6,7,8]. However, the demodulation technology of optical fiber sensors still often uses an optical spectrum analyzer (OSA) as the detection instrument, which has a limited resolution of 0.02 nm (2.5 GHz) [9]. The resolution of the OSA makes it difficult to detect the change in frequency below 1 GHz. Considering that the minimum resolution of the system depends on the sensitivity under the same experiment instrument, a high-sensitivity temperature sensor can improve the resolution for measurement by utilizing microwave photonic technology. Microwave photonics is a field that combines optical waves with microwaves for the generation, transmission, processing, and measurements of microwave signals by means of photonics, to take advantage of the high speed and broad bandwidth offered by modern photonics [10]. The electrical spectrum analyzer and the high-speed oscilloscope can be used to detect the small frequency variation in a microwave photonic sensing system, so the resolution and demodulation speed can reach 10^−6^ °C and MHz/s, respectively [11]. Microwave photonic sensing demodulation techniques include optical carrier-based microwave interferometry (OCMI) [12], a microwave photonic filter (MPF) [13], an optoelectronic oscillator (OEO) [14], and a microring resonator (MRR) [15]. The OCMI and MPF have the unique advantages of a large bandwidth and reconfigurable sensing spectrum [16,17]. The OEO sensing system has the capability of high-speed detection, and the MRR sensors can be used for miniaturized, integrated, high-sensitivity sensing [18,19]. In order to further improve the sensitivity of the microwave photonic sensing system, researchers have developed a slightly detuned interference frequency amplification technology called the Vernier effect [20,21,22].

In some optical measurements, the Vernier effect is an amplification process of the small change in the free spectral range (FSR), much like a Vernier caliper [23]. The Vernier effect amplifies slightly detuned interference frequencies between two comb signals with similar frequency intervals, which are implemented by two optical resonators with two similar FSRs, with one serving as a reference unit called FSR1 and the other as a sensing unit called FSR2 [24]. The Vernier effect can enhance the sensitivity of the system by detecting the shift in the amplified envelope curve generated by the overlap between FSR1 and FSR2, which has been applied for measuring temperature [25], pressure [26], and the refractive index [27]. The sensitivity of the Vernier effect is limited to the difference between the two FSRs. The smaller the difference between the two FSRs, the higher the sensing sensitivity. However, the traditional optical Vernier effect amplification measurements can only change the FSR of the sensing unit, and the other resonator often plays the reference role of which the FSR remains unchanged [28,29,30,31]. If the sensing parameters can be applied to the two resonators, one FSR increases, and the other FSR decreases at the same time, then an enhanced Vernier effect [27] can be achieved to further improve the sensing sensitivity. However, the enhanced Vernier effect usually use Fabry–Perot interferometers (FPIs) or other complex structures to be accomplished, and the sensing physical parameters are mostly related to displacement [32,33]. Those sensors with similar structures applied by temperature make it difficult to obtain two sensing curves of the FSR shift in different directions. Therefore, the change in temperature usually only increases or decreases the FSR of both resonators, and the sensitivity of the Vernier effect will not increase because the difference between the two FSRs remains unchanged. To further improve the temperature sensitivity based on the enhanced Vernier effect, we propose cascaded fiber loop interferometers that can make one FSR increase and the other decrease by designing the effective length difference between the three main interference paths.

In this work, to improve the change in the difference between two FSRs of the traditional Vernier effect sensing system, cascaded fiber loop interferometers are demonstrated to make the two spectra of FSRs shift in different directions, resulting in an enhanced Vernier effect. The sensing system incorporates a single-mode fiber and two fiber ring resonators, and they form an OCMI-based sensing system with three-beam interference. The length differences between each interference beam are similar, forming a Vernier effect spectral response. By changing the effective length of the sensing fiber loop, the path-length difference in one interference increases, and the other decreases. Thus, the FSR1 decreases, the FSR2 increases simultaneously, and the change in the difference between the two FSRs can be improved, achieving an enhanced Vernier effect. Experimental results demonstrate that the temperature sensitivity of a traditional Vernier effect measurement is only −298.29 kHz/°C, while our proposed enhanced Vernier effect sensing system achieves a sensitivity of 618.14 kHz/°C, which is 92 times higher than that of a two-arm optical carrier-based microwave interferometry sensing system and 2.07 times higher than that of a traditional Vernier effect sensing system, consistent with the theoretical predictions. The enhanced Vernier effect scheme based on cascaded fiber loop interferometers can be used to design high-sensitivity and integrated temperature sensors.

## 2. Principle

### Principle of OCMI Sensing System

**Figure 1:**
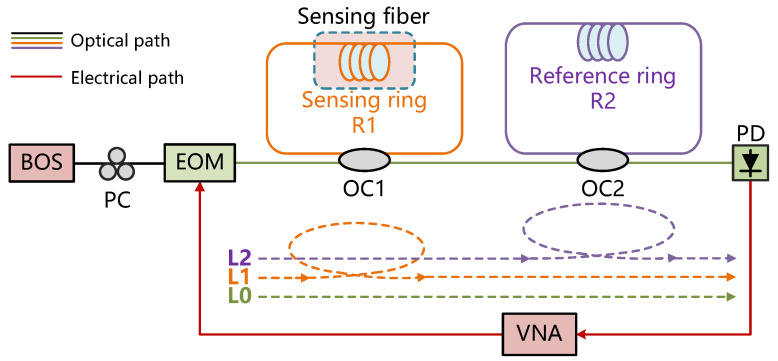
The schematic of the high-sensitivity temperature sensing system based on the enhanced Vernier effect and cascaded fiber loop interferometers. (BOS: broadband optical source; PC: polarization controller; EOM: electrooptical modulator; OC: optical coupler; R1: length of optical fiber loop 1; R2: length of optical fiber loop 2; PD: photodetector; and VNA: vector network analyzer).

Figure 1 shows the schematic of the proposed temperature sensing system. The broadband optical source (BOS) is used as the probe light. The polarization controller (PC) is used to control the polarization state. A generated microwave signal is applied to an electro-optical modulator (EOM), which is used for modulation. The probe light is divided by the optical coupler 1 (OC1) and the optical coupler 2 (OC2). Due to the large attenuation of light that circulates multiple times within the loop, three main interference paths are examined, designated as L0, L1, and L2, respectively. Three light waves with different time delays are injected into the photodetector (PD) and the vector network analyzer (VNA) for signal analysis. An OCMI sensing system with three-beam interference is thus established.

First, we consider the case where only R1 exists. The OCMI system corresponds to a two-tap microwave photonic filter of which the spectral response can be expressed as follows:

(1) $$\left|S_{21}\left(\omega \right)\right|=2G\left|\cos\left(\frac{\omega \Delta \tau }{2}\right)\right|$$

where *G* is the gain factor of the transmission system, including the optoelectronic conversion efficiency, the gain of the electrical amplifier, and the loss of the optical link; ω is the angular frequency of the microwave signal injected into the EOM, and $\Delta \tau =n_{eff}\Delta L/c$ is the time delay difference between the Mach–Zehnder Interferometer (MZI) reference arm and the sensing arm, where ΔL is the fiber length difference between the reference arm and the sensing arm, $n_{eff}$ is the effective refractive index of the fiber, and *c* is the speed of light in vacuum.

The free spectrum range of the two-tap OCMI sensing system is given by

(2) $$FSR=\frac{c}{n_{eff}\cdot \Delta L}$$

When the two-tap OCMI system is used for temperature sensing, the effective refractive index or the physical length difference between the two interferometer arms is varied, resulting in a varied FSR given by

(3) $$\begin{array}{ll} \Delta FSR & =\frac{c}{n_{eff}\cdot \Delta L+l\cdot \left(\xi \Delta T+\alpha \Delta T\right)}-\frac{c}{n_{eff}\cdot \Delta L}\\ & \approx FSR\cdot \frac{-l\cdot \left(\xi +\alpha \right)\Delta T}{n_{eff}\cdot \Delta L} \end{array}$$

where ΔFSR represents the change in the FSR affected by temperature changes; $\Delta n_{eff}$ represents the change in the effective refractive index affected by temperature; l indicates the fiber length for sensing; ΔT represents temperature change; $\xi \approx 8.5\times 10^{-7}\;{}^{\circ}C$ represents the thermo-optical coefficient of silicon dioxide material, and $\alpha \approx 5.5\times 10^{-6}\;{}^{\circ}C$ is the coefficient of thermal expansion [13]. The sensitivity can thus be enhanced by increasing the length of the sensing fiber and reducing the length difference between the two arms, or by increasing the conversion of the sensing temperature and the original free spectrum range.

**Figure 2:**
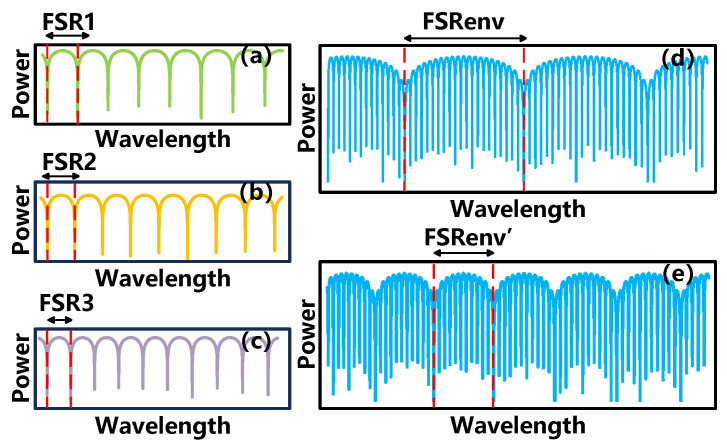
Optical Vernier effect envelope spectrum generated by superposition of different FSRs: (**a**) FSR1. (**b**) FSR2. (**c**) FSR3. (**d**) Superposition of FSR1 and FSR2. (**e**) Superposition of FSR1 and FSR3.

Then, R2 is incorporated, resulting in a three-tap OCMI sensing system with two optical fiber loop interferometers. Since the delay times between the three taps are similar, the filter spectrum with the Vernier effect can be generated. Figure 2 shows the envelope spectrums of the optical Vernier effect generated by the combinations of two similar FSRs. Figure 2a–c represents the three responses of different resonators, arranged from largest to smallest according to FSRs. The spectrums of FSR1 and FSR3 are combined, and a superposition of the Vernier effect spectrum corresponding to $FS{R^{\prime }}_{env}$ is generated in Figure 2e. When the FSR3 is increased to match FSR2, the combination of FSR1 and FSR2 spectrums, due to their closer proximity, will generate $FSR_{env}$, characterized by a larger envelope in Figure 2d.

The envelope relation of the three-tap OCMI system formed by superposition between two FSRs can be written as

(4) $$FSR_{env}=\left|\frac{FSR_{1}\times FSR_{2}}{FSR_{1}-FSR_{2}}\right|$$

where $FSR_{env}$ represents the width of the overall envelope spectrum. Equation (4) is the key derivation principle based on the optical Vernier effect. The magnification of the optical Vernier effect is defined as the ratio of the $FSR_{envelope}$ and the sensing FSR2. The magnification can then be expressed as

(5) $$M=\frac{FSR_{env}}{FSR_{2}}=\left|\frac{FSR_{1}}{FSR_{1}-FSR_{2}}\right|$$

According to Equation (5), when the value of FSR1 remains constant, the magnification factor M primarily depends on the difference in each FSR. The closer the physical parameters of the two optical resonators are, the greater the magnification of the sensitivity.

We define Δ to represent the change variation in the FSR due to the change in environmental parameters. It can be written as

(6) $$M^{i}=\left|\frac{FSR_{1}}{FSR_{1}\left(1-\Delta \right)-FSR_{2}\left(1+\Delta \right)}\right|\approx \left|\frac{FSR_{1}}{FSR_{1}-FSR_{2}\left(1+2\Delta \right)}\right|=2M$$

Therefore, the enhanced Vernier effect yields a sensitivity improvement that is twice that of traditional Vernier sensing schemes. The high-sensitivity enhanced Vernier effect temperature sensing system allows for the simultaneous adjustment of both resonators.

## 3. Experiment

Experiments were performed to verify the reliability of the enhanced Vernier effect system for temperature sensing, as illustrated in Figure 1. An amplified spontaneous emission (ASE730, THORLABS, Newtown, CT, USA) source with a 10 dB spectral width of 70 nm was used as the broadband optical source. The EOM (MX-LN-40, Photline Technologies, Besancon, France) has a bandwidth of 30 GHz, and the PC was utilized for controlling the polarization state. The coupling ratios of OC1 and OC2 are 50:50. The bandwidth of the PD (PD-50-S-FA-V, Realphoton, Shenzhen, China) is 30 GHz. The measurement range of the VNA (N5222A, Agilent Technologies, Santa Clara, CA, USA) is 10 MHz to 26.5 GHz. The system utilizes two commercial single-mode fibers for traditional and enhanced Vernier effect sensing, with lengths of 51.4 m (R1) and 104 m (R2), respectively. Since L0 serves as a common segment for all three main interference paths, its length can be adjusted flexibly. Consequently, the coherence length difference between L0 and L1 is set to 51.4 m, and the coherence length difference between L1 and L2 is set to 52.6 m. These close length differences are critical for generating an optical Vernier effect.

**Figure 3:**
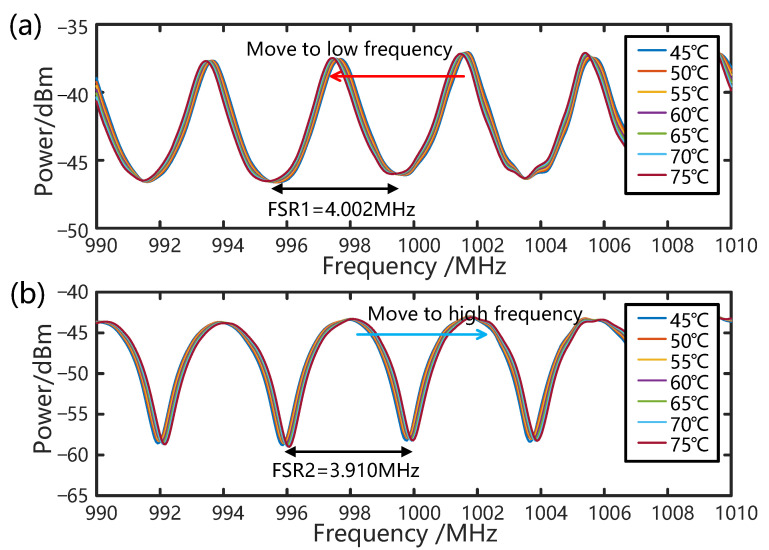
FSR change diagram of the two-beam OCMI sensing system as a response to temperature change: (**a**) FSR1 of L0 and L1. (**b**) FSR2 of L1 and L2.

Initially, typical OCMI-based temperature sensing experiments are performed by heating the sensing loop with a length of 49 m in a water bath where the temperature increases from 45 °C to 75 °C with an increment of 5 °C. Figure 3 shows the frequency response to temperature changes using only two paths, L0 and L1, and L1 and L2, respectively, which represent an OCMI-based sensing system with two-beam interference. Experimental results indicate that the FSR1 of paths L0 and L1 is 4.002 MHz, which aligns closely with the theoretical value of 4.008 MHz. Similarly, the FSR2 of paths L1 and L2 is 3.910 MHz, closely matching the theoretical value of 3.917 MHz. The theoretical FSRs are calculated when the fiber length differences between the two paths are 51.4 m and 52.6 m, respectively, and the effective refractive index of fiber measured by two-beam interferometry is 1.456. As shown in Figure 3a, when the temperature of L1 increases, the effective length difference between L0 and L1 also increases, resulting in a decrease in FSR1 and a shift in the resonant frequency towards lower values. Conversely, as the temperature of L1 increases, the effective length difference between L1 and L2 decreases, leading to an increase in FSR2 and a shift towards higher values, as illustrated in Figure 3b.

**Figure 4:**
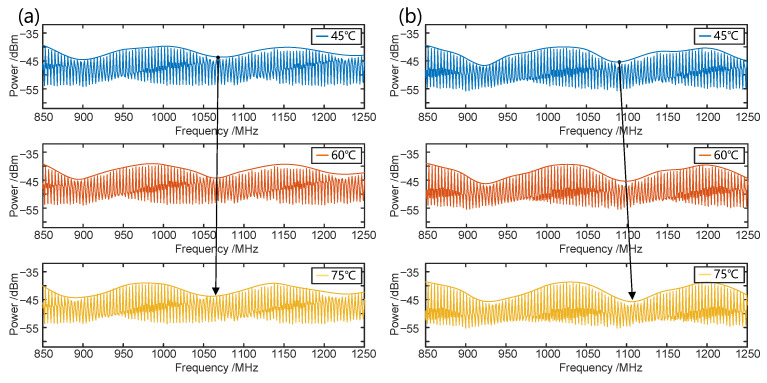
Two kinds of the Vernier effect temperature sensing system of envelope spectrum shifting: (**a**) The traditional scheme. (**b**) The enhanced scheme.

**Figure 5:**
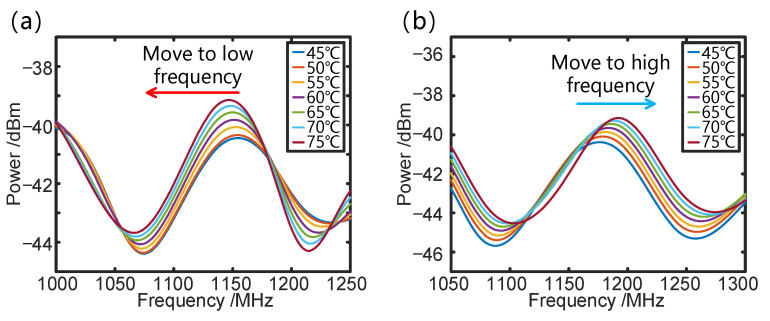
Envelope offset curves of the Vernier effect as the temperature changes: (**a**) Traditional test scheme. (**b**) Enhanced test scheme.

Then, we tested the enhanced temperature sensitivity of the proposed three-beam OCMI-based sensing system with cascaded fiber loop interferences. The 49 m fiber of R1 is placed in a water bath for enhanced Vernier effect sensing where the temperature increases from 45 °C to 75 °C with an increment of 5 °C. Under the same measurement conditions, we place an identical length of the R2 fiber in the water bath for a comparison experiment based on traditional Vernier effect temperature sensing. Figure 4a,b illustrates the Vernier effect spectra shift associated with the traditional Vernier effect and the enhanced Vernier effect when the temperature is set at 45 °C, 60 °C, and 75 °C. The arrow indicates the shifting direction of the envelope spectrum. As the temperature increases, the envelope curve under a traditional Vernier effect in Figure 5a shifts towards the low-frequency direction, whereas the envelope curve under the enhanced Vernier effect in Figure 5b shifts towards the high-frequency direction. It is evident that the envelope shift in the enhanced Vernier effect is larger than that of the traditional Vernier effect under the same temperature variation in Figure 5.

**Figure 6:**
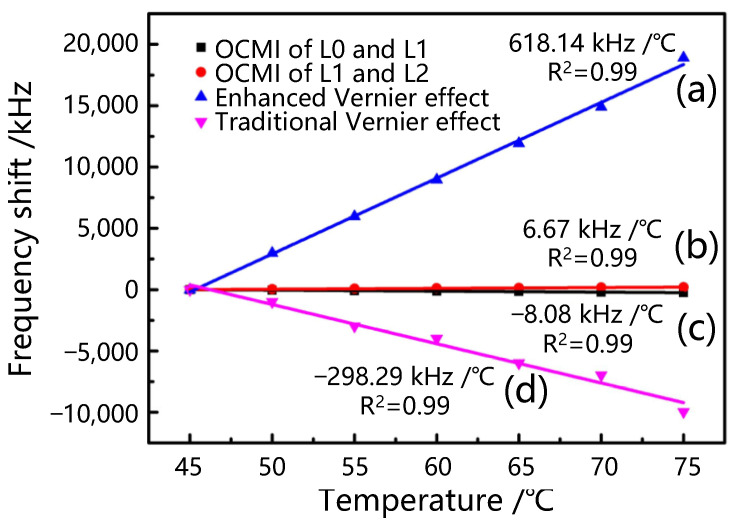
Linear fittings of temperature change and frequency shifts: (**a**) Enhanced Vernier effect. (**b**) L1 and L2. (**c**) L0 and L1. (**d**) Traditional Vernier effect.

Figure 6 illustrates the temperature sensitivity of four different test results: the OCMI of L0 and L1, the OCMI of L1 and L2, the enhanced Vernier effect, and the traditional Vernier effect. The sensitivity of paths L1 and L2 is 6.67 kHz/°C @1 GHz in Figure 6b, while the sensitivity of paths L0 and L1 is −8.08 kHz @1 GHz in Figure 6b. A sensitivity of −298.29 kHz/°C @1 GHz is obtained from the traditional Vernier effect with the three-beam interference in Figure 6d, whereas the sensitivity of our enhanced optical Vernier effect can reach 618.14 kHz/°C @1 GHz in Figure 6a, which is 2.07 times higher than that of the traditional Vernier effect scheme and 92 times larger than that of the ordinary OCMI system with two-beam interference, consistent with the theoretical predictions according to Equation (6).

**Figure 7:**
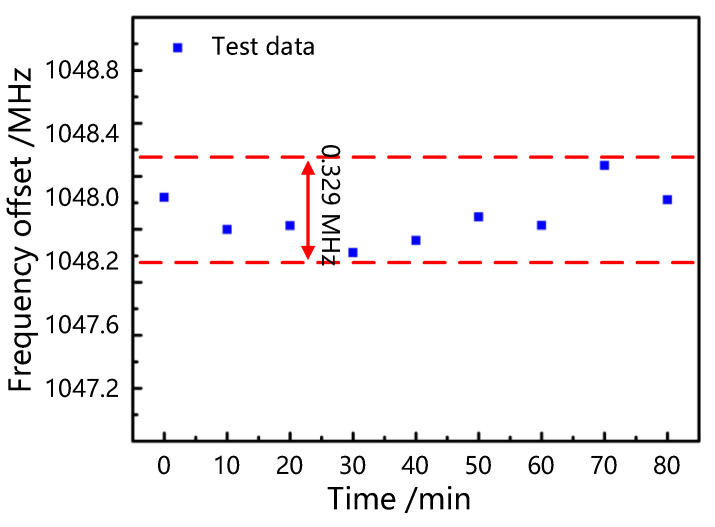
Stability analysis diagram of temperature sensing system based on enhanced Vernier effect.

The sensing loop is placed at 25 °C for 80 min, and the sampling interval is 10 min to assess the sensor system’s stability. Figure 7 illustrates the stability test date of our cascade fiber loop temperature sensing system based on the enhanced Vernier effect. The blue points are experimental data, and the red dashed lines indicate the jitter range of the frequency. When the test frequency is 1 GHz, the maximum variation in the frequency is about ±0.329 MHz. This is due to the limited accuracy of the water bath and the disturbance of the sensing fiber vibrations. The stability can be greatly improved by encapsulating the fiber sensor and utilizing a more accurate temperature control platform in real applications.

Table 1 lists the sensitivity of some temperature sensors based on the Vernier effect and microwave photonic sensing technology in the frequency domain. The sensitivity of all sensing structures should be standardized to the same detection frequency for comparison, considering that the sensitivity of sensors depends on the measurement frequency. The proposed temperature sensing system in this work with an enhanced Vernier effect has the highest sensitivity compared to other reported approaches. A sensitivity of 618.14 kHz/°C at 1 GHz was obtained in this work, which is not only larger than the sensitivity of the traditional Vernier effect works, but also larger than the sensitivity of the reported enhanced Vernier effect works.

## 4. Conclusions

In conclusion, we propose a high-sensitivity temperature sensing system that generates an enhanced Vernier effect using cascaded fiber loop interferometers to form a three-beam OCMI-based interferometer, demonstrating its applicability to temperature sensing through experimental validation. The enhanced Vernier effect is produced by improving the change in the difference between the two FSRs. By changing the effective length of fiber loop 1, the FSR1, generated by L0 and L1, decreases, while the FSR2, generated by L1 and L2, increases simultaneously, resulting in an enhanced Vernier effect. In the same condition, the sensitivity of the two-arm OCMI sensing system is only 6.67 kHz/°C @ 1 GHz, and the traditional Vernier effect is −298.29 kHz/°C @ 1 GHz, whereas the temperature sensitivity of our proposed sensing system can reach 618.14 kHz/°C @ 1 GHz, which is not only a 92-fold increase over the sensitivity of the two-arm OCMI sensing system but also doubles that of the traditional Vernier effect. The proposed sensing scheme is characterized by an enhanced Vernier effect and can therefore be applied for high-sensitivity temperature measurements. This work provides an idea for integrated structure and holds potential applications in biometrics, smart cities, and the Internet of Things.

## Figures and Tables

**Table 1:** Comparison of several temperature sensing systems based on microwave photonic technology and optical Vernier effect measurement schemes.

Sensing Structure	Vernier Effect	Sensing System	Sensitivity@ 1 GHz (kHz/°C)	Ref.
Cascaded fiber loop	Traditional	MPF	−230.25	[28]
Positive and negative dispersion fiber	Enhanced	OEO	−68.18	[34]
Polarization multiplexing	Traditional	OEO	−402.5	[29]
Frequency comb and fiber ring	Traditional	MPF	−140.178	[30]
Parallel MZIs	Enhanced	MPF	395.84	[35]
Cascaded MZIs	Traditional	MPF	290.225	[31]
Cascaded FPIs	Traditional	MPF	185.3	[36]
Cascaded fiber loop	Enhanced	MPF	618.14	This work

## Data Availability

Data are contained within the article.
